# Economic evaluation of three forms of early intervention for young people with borderline personality disorder: a within-trial cost-utility analysis from the MOBY clinical trial

**DOI:** 10.1007/s00787-026-02994-9

**Published:** 2026-03-16

**Authors:** Yong Yi Lee, Jennifer K. Betts, Ellen Lardner, Henry Jackson, Sue M. Cotton, John Gleeson, Christopher G. Davey, Sharnel Perera, Victoria Rayner, Louise McCutcheon, Cathrine Mihalopoulos, Andrew M. Chanen

**Affiliations:** 1 School of Public Health and Preventive Medicine, Monash University Health Economics Group, Monash University, Melbourne, Australia; 2 School of Public Health, The University of Queensland, Brisbane, Australia; 3 Queensland Centre for Mental Health Research, Brisbane, Australia; 4 Orygen, Melbourne, Australia; 5 Centre for Youth Mental Health, The University of Melbourne, Melbourne, Australia; 6 Deakin Health Economics, School of Health and Social Development, Deakin University, Melbourne, Australia; 7 Melbourne School of Psychological Sciences, The University of Melbourne, Melbourne, Australia; 8 School of Psychological Sciences, Monash University, Melbourne, Australia; 9 Healthy Brain and Mind Research Centre, School of Behavioural and Health Sciences, Australian Catholic University, Melbourne, Australia; 10 Department of Psychiatry, The University of Melbourne, Melbourne, Australia

**Keywords:** Cost-effectiveness, Randomised controlled trial, Early intervention, Borderline personality disorder, Youth, Psychiatry

## Abstract

Borderline personality disorder (BPD) is a severe mental disorder that begins in adolescence and emerging adulthood. This study evaluated the comparative cost-effectiveness of three forms of early intervention, each with differing levels of treatment complexity, for young people with recently-diagnosed BPD. A cost-utility analysis was conducted alongside the Monitoring Outcomes of BPD in Youth (MOBY) randomised controlled trial involving 139 Australian participants, aged 15–25 years, who were diagnosed with BPD. The three MOBY treatment arms (from highest-to-lowest treatment complexity) were: (1) HYPE + CAT, the Helping Young People Early (HYPE) dedicated BPD service model for young people, combined with weekly Cognitive Analytic Therapy; (2) HYPE + Bef, HYPE combined with a weekly befriending psycotherapy control condition; and (3) YMHS + Bef, a general youth mental health service model, combined with Befriending. Cost and outcomes data were collected over 18 months, with costs denominated in 2015 Australian dollars (Ah$), and quality-adjusted life year (QALY) outcomes derived using the Assessment of Quality of Life-8D questionnaire. Cost-effectiveness results were expressed as an incremental cost-effectiveness ratio (ICER). There were no significant differences between the three treatment arms in costs or QALYs. The three treatment arms demonstrated similar levels of cost-effectiveness to one another. Under a willingness-to-pay threshold of A$96,000 per QALY, the probability of each intervention being cost-effective (when assuming mutual exclusivity) was: 36.4% for HYPE + CAT; 30.9% for HYPE + Bef; and 32.7% for YMHS + Bef. For young people with recently-diagnosed BPD, and who have never received evidence-based BPD treatment, early intervention has the potential to represent good value for money. Both specialist and generalist service models appear to be similarly cost-effective.

**Supplementary Information:**

The online version contains supplementary material available at 10.1007/s00787-026-02994-9.

## Introduction

Borderline personality disorder (BPD) is a severe mental disorder that is characterised by a pervasive pattern of instability of interpersonal relationships, self-image, emotions and impulsivity [1]. BPD is associated with significant and widespread reductions in quality of life across the mental, social and physical domains [2, 3], having among the most severe impairments of all mental disorders [4, 5]. Compared with the general population, people with BPD have an 8-fold increase in all-cause mortality [6] and a 45-fold increase in death by suicide [7]. They also have a reduced life expectancy of up to two decades [8, 9]. The societal costs of BPD are also substantial, with a Danish national registry data study finding a 16-fold increase in healthcare costs and lost productivity due to poor health compared with matched controls [5].

Despite having its clinical onset between puberty and young adulthood, the opportunity to reduce or avoid adverse outcomes associated with BPD is routinely missed due to delays in diagnosis and treatment [10]. While a range of specialised psychosocial treatments for BPD in young people have been studied, they have shown modest effectiveness [11, 12] and few trials have focused on early-stage disorder. Monitoring Outcomes of Borderline Personality Disorder in Youth (MOBY) [13] was the first randomised controlled trial (RCT) to enrol only young people with early stage, full-syndrome BPD who had never received evidence-based BPD care. MOBY investigated the effectiveness of three early interventions for BPD of differing complexity [13]. This Australian RCT found that, regardless of group, from baseline to 12 months there was a mean of 19.3% and 23.8% improvement in the joint primary outcomes of social adjustment and interpersonal problems (‘psychosocial functioning’), respectively. Improvement in the seven secondary outcomes ranged from 40.7% (depression) to 52.7% (suicidal ideation), except for severity of substance use and client satisfaction. The latter remained high across all time points. Furthermore, neither the type of service model nor the inclusion of an individual psychotherapy intervention was associated with a superior rate of change in psychosocial functioning by the 12-month primary endpoint [13]. The specialist service model (versus generalist) and the individual psychotherapy (versus non-psychotherapy control) were found to be superior with regard to treatment attendance and completion. Taken together, the findings suggest that a dedicated service model, with or without psychotherapy, might strike the right balance of services to best meet service user, family and carer expectations of care.

Several economic evaluations have examined the costs and cost-effectiveness of treating BPD pathology [14–16]. Only one within-trial evaluation has focused exclusively on young people [17], but this trial only studied adolescents (aged 12–18 years) and only 20.5% of participants were diagnosed with the full BPD syndrome. No economic evaluation has either analysed the cost-effectiveness of early intervention for young people with a recent diagnosis of BPD, or has identified which components of early intervention might be considered value-for-money in this target population. To address this evidence gap, the current study presents the findings of an economic evaluation, conducted alongside the MOBY trial, examining the comparative cost-effectiveness of three forms of early intervention for young people with recently-diagnosed BPD.

## Methods

### Intervention trial

A within-trial economic evaluation was conducted alongside the MOBY trial [13]. Study participants were help-seeking young people: aged 15–25 years; with a recent DSM-IV-TR diagnosis of BPD; with no DSM-IV-TR psychotic or bipolar disorder diagnosis; and who had never received an evidence-based BPD treatment. The three treatment arms were: (1) HYPE + CAT – the Helping Young People Early (HYPE) service model, which is a dedicated, specialised BPD service model combined with weekly individual Cognitive Analytic Therapy (CAT); (2) HYPE + Bef – the HYPE service model combined with weekly individual befriending (Bef), a psychotherapy-control condition where participants spend time discussing neutral topics of interest (e.g. sport, music, work); and (3) YMHS + Bef – a general youth mental health service (YMHS) model that was implemented by mental health clinicians who had expertise in treating young people, but who were not BPD specialists, combined with befriending. The three treatment arms represented different levels of treatment complexity defined by combining either a specialist or a generalist service delivery model (HYPE versus YMHS) with either an individual psychotherapy or a control psychotherapy condition (CAT versus Bef). The two HYPE study arms were implemented at Orygen, while YMHS was implemented at a *headspace* centre. Orygen and *headspace* are government-funded youth mental health services, located in Melbourne, Australia. The study protocol and analyses of clinical outcomes are described elsewhere [13, 18].

### Cost analysis

This economic evaluation adopted an Australian healthcare sector perspective, which encompassed costs involving: (1) the direct cost of delivering each of the three MOBY interventions (HYPE + CAT, HYPE + Bef and YMHS + Bef); (2) the cost of other community-based healthcare services accessed by study participants over the MOBY trial period from randomisation to the 18-month secondary endpoint; (3) the cost of psychotropic medications used during the trial period; and (4) the cost of emergency department presentations and hospital admissions that occurred during the trial period. Detailed methods used to derive each cost type are described in Appendix S1. Each treatment arm was evaluated separately and all costs were denominated in 2015 Australian dollars (A$). The 2015 reference year corresponded with the final year that participants were enrolled in the MOBY trial. An 18-month time horizon, aligning with the timeframe of the MOBY trial, was used. Discounting (a method used to adjust costs to account for the higher value that people place on money obtained in the present, compared with the future) was not applied in the base case analysis due to the short study timeframe (~ 1.5 years).

### Health outcomes

Changes to participants’ health-related quality of life were measured using the Assessment of Quality of Life-8D (AQoL-8D) [19] at baseline and again at the 3-, 6-, 12- and 18-month time points. Australian general population preference weights (for those aged ≥ 16 years) were applied to calculate utility weights at each time point [20]. The utility weight is a summary measure of health status that comprises a numeric value between 0 (denoting death) and 1 (denoting perfect health). The area-under-the-curve (AUC) method was used to convert utility weights into quality-adjusted life years (QALYs).

### Statistical analysis

This study adheres to the Consolidated Health Economic Evaluation Reporting Standards (CHEERS) and has a completed impact inventory, as recommended by the Second Panel on Cost-Effectiveness in Health and Medicine (see Appendix S2). Statistical analyses were conducted using Stata 17 (College Station, Texas, USA). Researchers analysing costs and health outcomes were not blinded to treatment arm allocation. The base case analysis adopted a healthcare sector perspective (to focus the analysis on costs and health outcomes accruing within the healthcare sector, while excluding broader societal impacts in the general economy – e.g., impacts on job productivity and education), alongside the intention-to-treat approach. All 139 enrolled participants were included and retention rates in the research assessments did not differ significantly between the three treatment arms at any time point [13]. Data on costs and AQoL-8D utility weights were observed to be missing across multiple follow-up periods. For example, at the 18-month follow-up period, a total of: 86 participants (61.9%) had complete utility data; and 70 participants (50.4%) had complete data on the cost of other healthcare services and psychotropic medications (see Appendix S3 for an analysis of missing data patterns). Multiple imputation methods were implemented in Stata to account for missing cost and utility weight data, under the assumption that these data were ‘missing at random’. This was done to leverage available data on costs and utility weights that would otherwise be omitted when analysing complete case data for total costs and total QALYs (see Appendix S3 for a detailed rationale behind this decision, alongside several exploratory analyses evaluating the appropriateness of the ‘missing at random’ assumption). In line with previous recommendations [21], a total of 50 imputed datasets were generated using multiple imputation by chained equations (MICE) with predictive mean matching and separate imputations stratified by treatment arm.

The difference in mean QALYs between treatment arms was estimated using a generalised linear model (GLM) (Gaussian family and identity link). The difference in mean costs accruing under the healthcare sector perspective was also estimated using a GLM (gamma family and log link). Baseline AQoL-8D utility weights were included as a baseline covariate for GLMs involving QALY outcomes [22]. All GLMs included adjustment for covariates involving the strata in randomisation – i.e., participants were stratified by age (cut point of age 18 years), sex assigned at birth and Center for Epidemiological Studies Depression Scale-Revised score (cut point of 37) [13].

Incremental cost-effectiveness ratios (ICERs) were estimated as the difference in mean costs between the intervention and comparator arms, divided by the difference in mean QALYs. Three sets of comparisons were analysed: (1) HYPE + CAT versus YMHS + Bef; (2) HYPE + Bef versus YMHS + Bef; and (3) HYPE + CAT versus HYPE + Bef. The second comparison directly contrasted the specialist HYPE service model to the generalist YMHS model, while the third comparison contrasted CAT against befriending. ICERs were calculated for each of the three comparisons. A resampling method comprising single imputation nested in bootstrapping was used to estimate 95% confidence intervals (95% CIs) around each ICER [23]. This method jointly accounts for variation due to sampling and missing data by generating a single call to the MICE procedure (outlined previously), which then produces a complete dataset by which to analyse GLMs of costs/QALYs within each bootstrap resample. A total of 3,000 bootstrap resamples were generated to produce a cost-effectiveness plane that graphically depicts the statistical dispersion surrounding estimates of the differences in mean costs/QALYs and resulting ICERs [24]. In the absence of an explicit Australian willingness-to-pay threshold (i.e., the ICER estimate below which a healthcare consumer would be prepared to pay for a health benefit) [25], interventions were judged to be cost-effective relative to a benchmark threshold of A$96,000 per QALY which was used in a recent Productivity Commission inquiry report on mental health (see p. 174 of Appendix I) [26]. ICERs situated above this threshold would be considered too expensive and not worth paying for. Cost-effectiveness acceptability curves were subsequently used to depict the probability of an intervention being cost-effective under different willingness-to-pay thresholds.

### Sensitivity analysis

A sensitivity analysis (SA) was performed to test the impact of conducting a complete case analysis involving the 44 participants with complete (non-missing) cost and QALY data. This sensitivity analysis examined how results would change when there was no multiple imputation of missing data.

## Results

### Intervention trial

Baseline demographic and clinical variables are presented in Appendix S4 for the total sample and across the three treatment arms. The majority of respondents were female and born in Australia, with 40% not in education, employment or training (NEET).

### Cost analysis

**Fig. 1:**
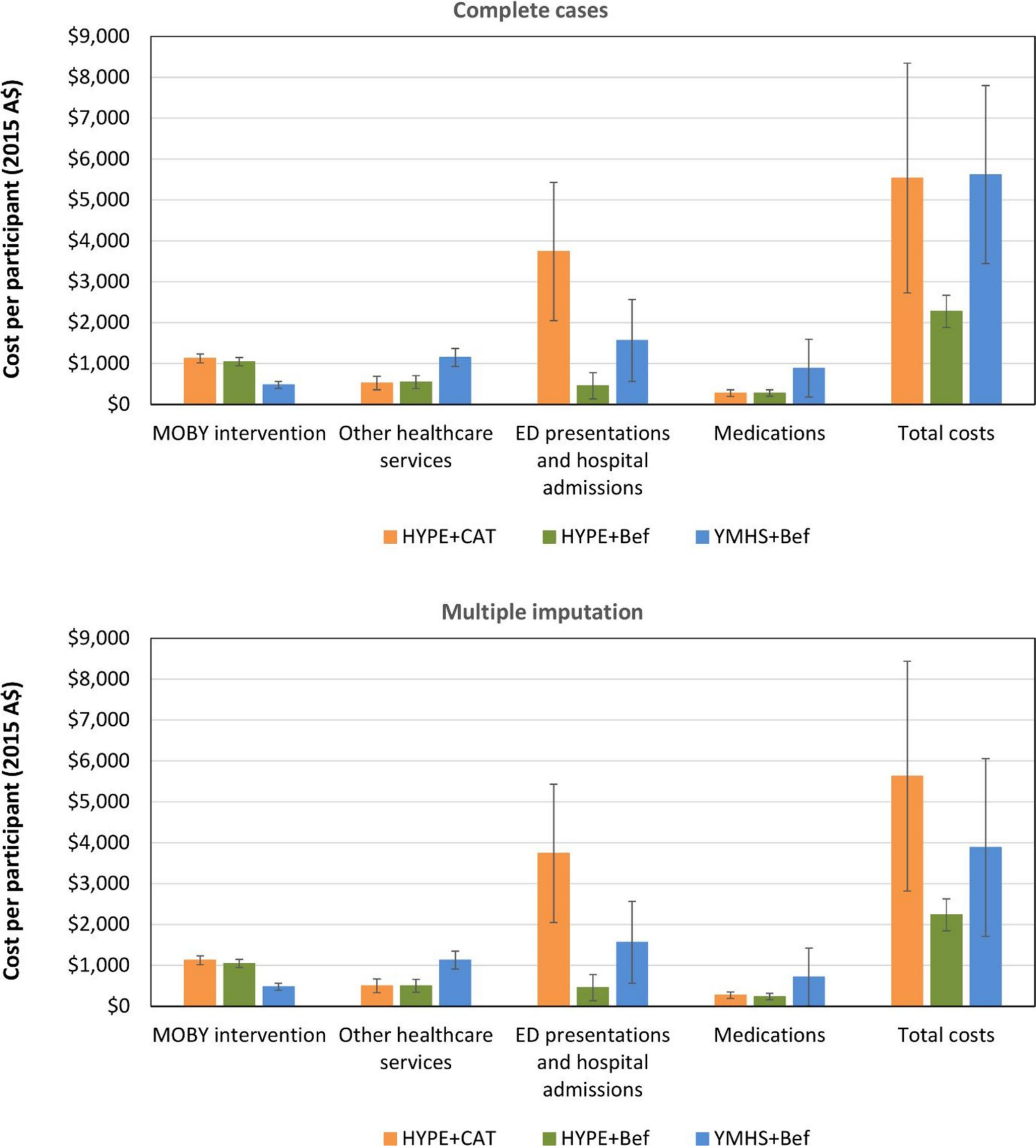
Mean cost per study participant across each of the three treatment arms (complete cases and multiple imputation). Abbreviations: A$ - Australian dollars; Bef - befriending; CAT - cognitive analytic therapy; ED - emergency department; HYPE - Helping Young People Early; YMHS - Youth Mental Health Service. Note: The standard error of each estimate is denoted by the error bars. Complete case data is available for all participants when analysing the cost per participant involving the MOBY intervention and ED presentations and hospital admission – i.e., HYPE + CAT (*n* = 46), HYPE + Bef (*n* = 46) and YMHS + Bef (*n* = 47). By comparison, complete case data is only available for a portion of participants when analysing the cost per participant involving Other healthcare services, Medications and Total costs – HYPE + CAT (*n* = 20), HYPE + Bef (*n* = 26) and YMHS + Bef (*n* = 24). Appendix S6 presents this data in full

Detailed unit costs for the MOBY intervention, other healthcare services, psychotropic medications, and emergency department presentations and hospital admissions are provided in Appendix S5. The mean total cost per person (estimated using multiple imputation) is shown for each of the three treatment arms in Fig. 1 and Appendix S6. The direct cost of delivering the MOBY intervention was highest among participants in the HYPE + CAT and HYPE + Bef groups ($1,123 [SE: 110] and $1,046 [SE: 100] per person, respectively), when compared with the YMHS + Bef arm ($476 [SE: 85]). This was due to HYPE participants attending a greater number of treatment sessions, compared with those in the YMHS service. The mean number of MOBY intervention sessions undertaken was: 19.9 (SE: 2.1) for HYPE + CAT; 18.3 (SE: 1.7) for HYPE + Bef; and 9.1 (SE: 1.6) for YMHS + Bef. The cost per MOBY intervention session, inclusive of CAT/Bef and other intervention sessions, was: $56 (SE: 8) for HYPE + CAT; $57 (SE: 8) for HYPE + Bef; and $52 (SE: 13) for YMHS + Bef. The largest contributor to total costs across each treatment arm involved emergency department presentations and hospital admissions – i.e., $3,740 per person (SE: 1,693) for HYPE + CAT, $454 (SE: 320) for HYPE + Bef and $1,564 (SE: 1,004) for YMHS + Bef. When examining all costs incurred over the 18-month period, the HYPE + CAT arm had the highest total cost at $5,629 per person (SE: 1,765); followed by the HYPE + Bef arm at $3,883 (SE: 1,211) and the YMHS + Bef arm at $2,236 (SE: 383). Incremental costs between treatment arms are shown in Table 1. The cost of HYPE + CAT was higher than the cost of YMHS + Bef but this difference was not statistically significant ($1,721, 95% CI: -2,202 to 5,775), while the cost of HYPE + Bef was higher than YMHS + Bef but this difference was not statistically significant (-$1,471, 95% CI: -4,086 to 573). By contrast, the cost of HYPE + CAT was significantly higher than the cost of HYPE + Bef ($3,192, 95% CI: 326 to 6,710).

**Table 1:** Cost-effectiveness results for each comparison set (presented by analytic method)

Comparison set (by analytic method)	Incremental costs, A$ (95% CI)	Incremental QALYs (95% CI)	Mean ICER, A$ per QALY (95% CI)
*HYPE + CAT vs. YMHS + Bef*			
Bootstrapping ^a^	$1,721 (-2,202 to 5,775)	0.018 (-0.121 to 0.152)	$95,633 (*Not defined* ^c^)
SA Complete case analysis ^b^	-$447 (-4,171 to 2,402)	0.071 (-0.128 to 0.246)	Dominant ^d^ (*Not defined* ^c^)
*HYPE + Bef vs. YMHS + Bef*			
Bootstrapping ^a^	-$1,471 (-4,086 to 573)	-0.015 (-0.140 to 0.108)	$96,970 ^e^ (*Not defined* ^c^)
SA Complete case analysis ^b^	-$1,651 (-5,587 to 792)	-0.088 (-0.278 to 0.085)	$18,810 (*Not defined* ^c^)
*HYPE + CAT vs. HYPE + Bef*			
Bootstrapping ^a^	$3,192 * (326 to 6,710)	0.033 (-0.088 to 0.158)	$96,244 (4,562 to Dominated ^f^)
SA Complete case analysis ^b^	$1,204 (-283 to 3,058)	0.158 (0.019 to 0.286)	$7,607 (Dominant ^d^ to 58,237)

Abbreviations: 95% CI − 95% confidence interval; A$ - Australian dollars; Bef - befriending; CAT - Cognitive Analytic Therapy; HYPE - Helping Young People Early; ICER - incremental cost-effectiveness ratio; QALYs - quality-adjusted life years; YMHS - Youth Mental Health Service

^a^ Single imputation nested within bootstrapping (3,000 iterations)

^b^ Complete case analysis with bootstrapping (3,000 iterations) and no imputation of missing data

^c^ 95% confidence interval is not defined as the ICER spans all four quadrants of the cost-effectiveness plane

^d^ A ‘dominant’ ICER indicates that the intervention costs less and is more effective than the comparator

^e^ ICER situated in the South-West quadrant of the cost-effectiveness plane – i.e., the HYPE + Bef intervention had a lower cost and produced less QALYs relative to YMHS + Bef. Larger ICERs in the South-West quadrant denote a more cost-effective intervention due to higher cost-savings relative to the QALY decrement

^f^ A ‘dominated’ ICER indicates that the intervention costs more and is less effective than the comparator

* Statistically significant at the 5% level of significance (*p* < 0.05)

### Health outcomes

**Fig. 2:**
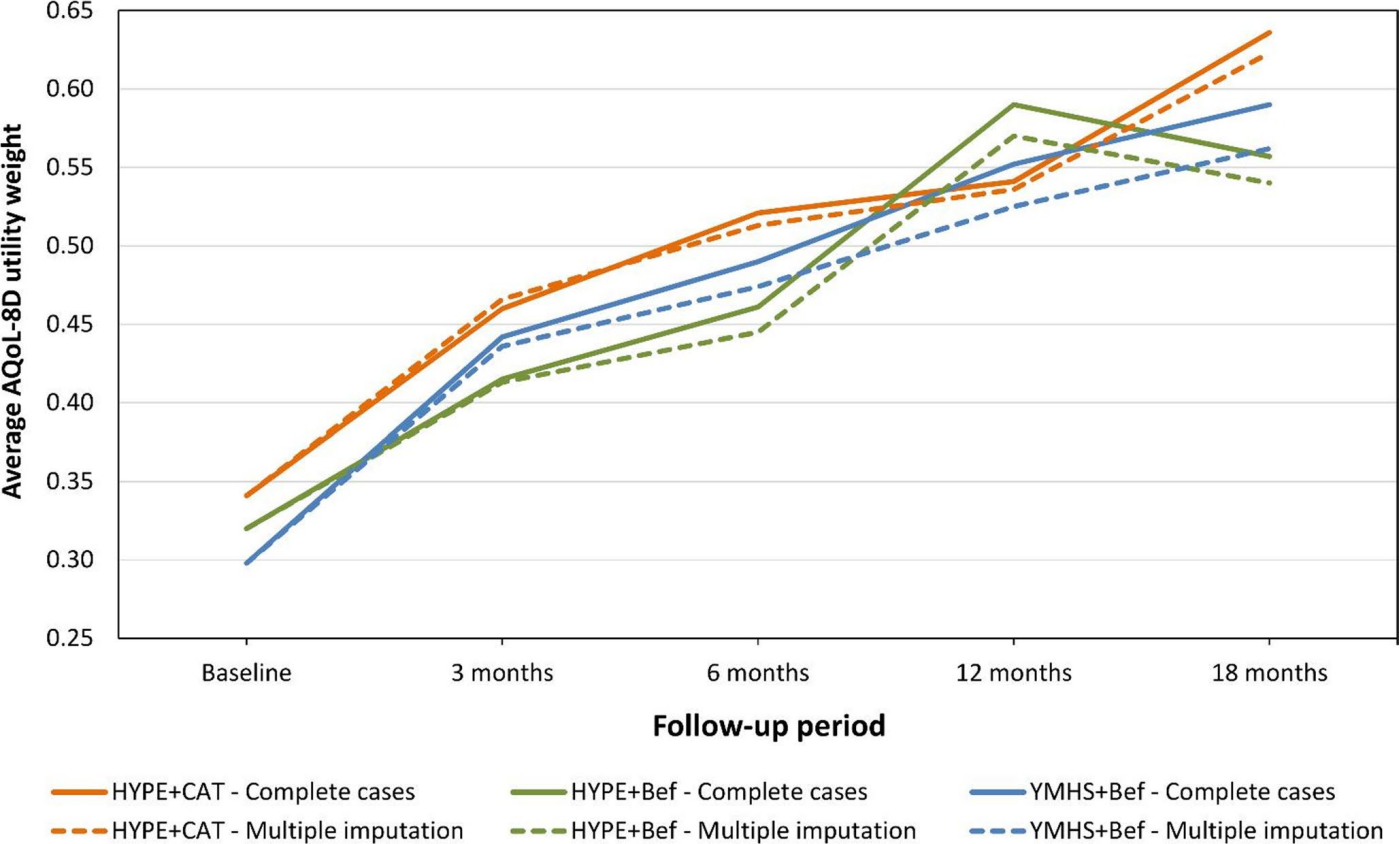
AQoL-8D utility weights for each of the three treatment arms by follow-up period (complete cases and multiple imputation). *Abbreviations: AQoL-8D - Assessment of Quality of Life-8D; Bef - befriending; CAT - Cognitive Analytic Therapy; HYPE - Helping Young People Early; YMHS - Youth Mental Health Service*

AQoL-8D utility weights at baseline and follow-up (estimated using multiple imputation) are presented in Fig. 2 and Appendix S6. Slight variations were observed between utility weights across the three treatment arms at baseline, though observed differences were not statistically significant – i.e., 0.341 (SE: 0.023) for HYPE + CAT, 0.320 (SE: 0.018) for HYPE + Bef and 0.298 (SE: 0.016) for YMHS + Bef. A trend toward increasing scores was also observed across each of the three treatment arms. For instance, utility weights at 18-month follow-up were 0.623 (SE: 0.043) for HYPE + CAT, 0.540 (SE: 0.038) for HYPE + Bef and 0.562 (SE: 0.044) for YMHS + Bef. The resulting incremental QALYs between HYPE + CAT and YMHS + Bef (see Table 1) were positive but not statistically significant (0.018, 95% CI: -0.121 to 0.152). Incremental QALYs between HYPE + CAT and HYPE + Bef were similarly positive but not significant (0.033, 95% CI: -0.088 to 0.158). Conversely, incremental QALYs between the HYPE + Bef and YMHS + Bef treatment arms were negative and not statistically significant (-0.015, 95% CI: -0.140 to 0.108).

### Cost-effectiveness results

**Fig. 3:**
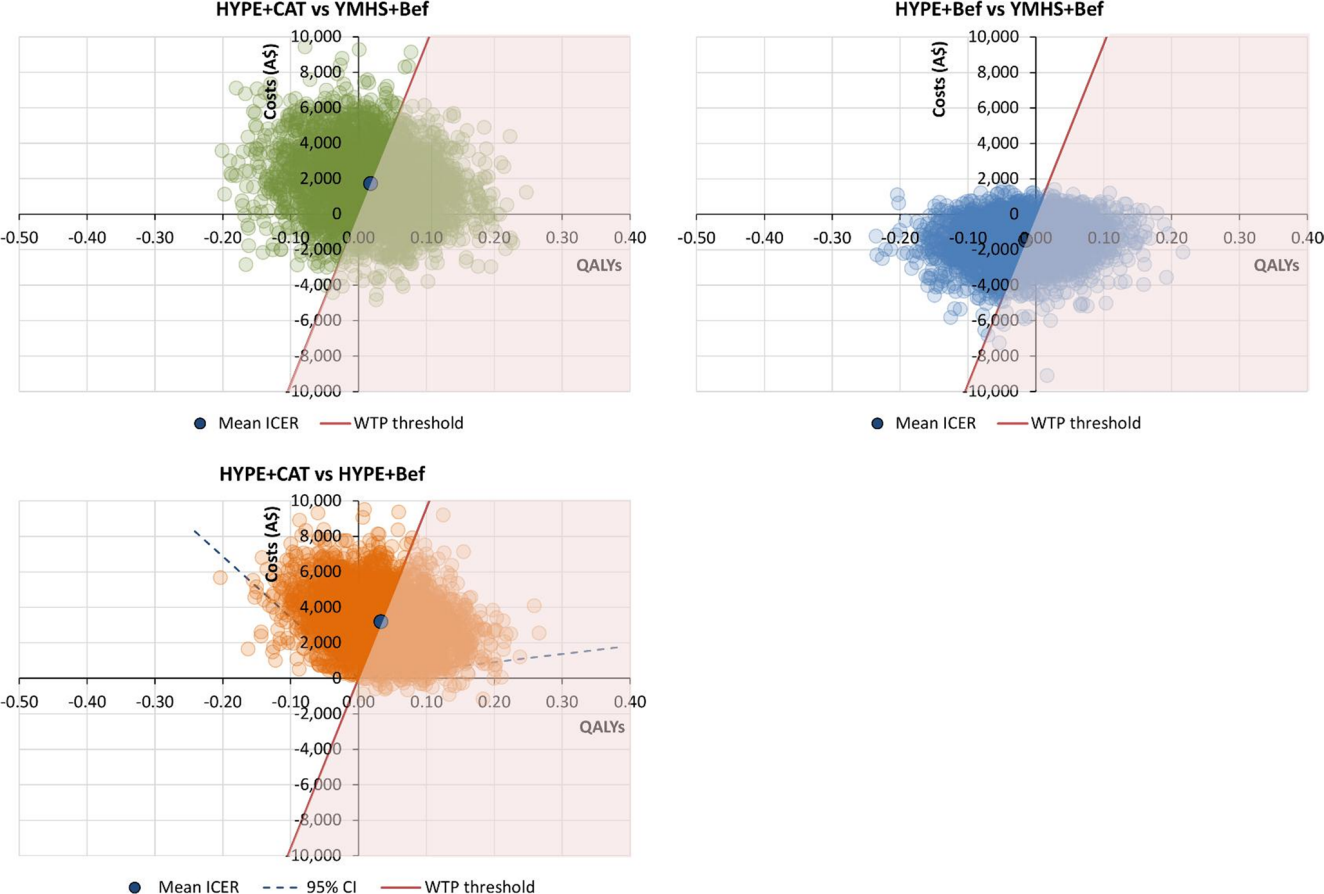
Cost-effectiveness planes for each comparison set (bootstrapping analysis). Abbreviations: 95% CI − 95% confidence interval; A$ - Australian dollars; Bef - befriending; CAT - Cognitive Analytic Therapy; HYPE - Helping Young People Early; ICER - incremental cost-effectiveness ratio; QALYs - quality-adjusted life years; WTP - willingness-to-pay; YMHS - Youth Mental Health Service. Note: The shaded area represents the region below the willingness-to-pay threshold where ICER estimates are considered cost-effective. ICER estimates above the threshold are considered too expensive

The comparison of HYPE + CAT versus YMHS + Bef produced a mean ICER of $95,633 per QALY that was situated in the North-East quadrant of the cost-effectiveness plane (see Table 1; Fig. 3), meaning that HYPE + CAT was more effective and more expensive than YMHS + Bef. Conversely, the comparison of HYPE + Bef to YMHS + Bef produced an ICER of $96,970 per QALY that was situated in the South-West quadrant of the cost-effectiveness plane – i.e., the HYPE + Bef intervention had a lower cost and produced less QALYs relative to YMHS + Bef. Lastly, the mean ICER of HYPE + CAT versus HYPE + Bef ($96,244 per QALY) was situated in the North-East quadrant. Overall, the mean ICERs for all three comparison sets were observed to overlap with the willingness-to-pay threshold of $96,000 per QALY. This suggested that all three interventions were likely to be as cost-effective as one another. In Fig. 3, a high degree of uncertainty was observed around incremental QALYs across all three comparison sets. Bootstrap replicates spanned all four quadrants of the cost-effectiveness plane when comparing HYPE + CAT versus YMHS + Bef and HYPE + Bef versus YMHS + Bef. This indicated large uncertainty around mean ICERs, which led to 95% confidence intervals that could not be defined.

**Fig. 4:**
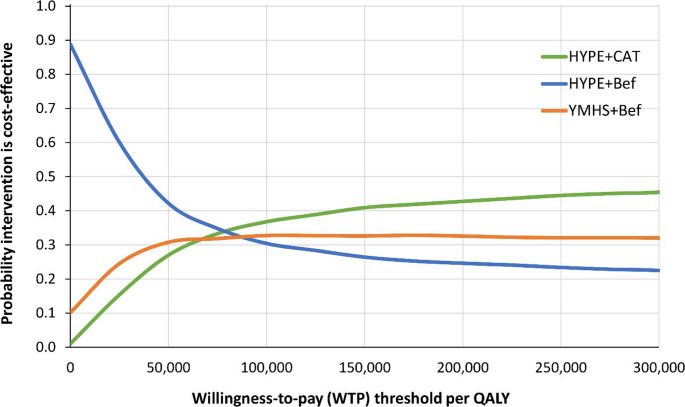
Cost-effectiveness acceptability curves for each of the three treatment arms. Abbreviations: Bef - befriending; CAT - Cognitive Analytic Therapy; HYPE - Helping Young People Early; QALYs - quality-adjusted life years; YMHS - Youth Mental Health Service

Cost-effectiveness acceptability curves are presented for each treatment arm in Fig. 4. The probability of these interventions being cost-effective was estimated by assuming that: (1) the three treatment options were mutually exclusive; and (2) a decision maker must choose to scale up one of these treatments for BPD (i.e., they cannot choose to do nothing). The probability of each treatment being cost-effective at an Australian willingness-to-pay threshold of A$96,000 per QALY was: 36.4% for HYPE + CAT; 32.7% for HYPE + Bef; and 30.9% for YMHS + Bef. Overall, these findings and the lack of significant differences in incremental costs/QALYs between treatment arms collectively suggest that all of the three interventions have similar likelihoods of being cost-effective, compared with one another. Specifically, no intervention was found to be superior to another in terms of cost-effectiveness.

### Sensitivity analysis

The complete case analysis (SA) shown in Table 1 led to substantive changes in mean ICERs; with HYPE + CAT and HYPE + Bef demonstrating much more favourable cost-effectiveness results when compared to YMHS + Bef. Figures 1 and 2 present how cost data and utility weights shifted across the complete case and multiple imputation analyses. It is likely that total costs and QALYs estimated in the complete case analysis were biased by missing data.

## Discussion

### Summary of findings

To the authors’ knowledge, this is the first economic evaluation of early intervention for young people with BPD. Three key findings emerged from this study. First, the only significant difference in costs between treatment arms was for the comparison between HYPE + CAT and HYPE + Bef. Second, the analysis of health outcomes demonstrated no significant differences in QALYs between treatment arms. Third, the cost-utility analysis demonstrated that none of the three treatments (HYPE + CAT, HYPE + Bef or YMHS + Bef) were found to be superior in terms of their cost-effectiveness credentials.

Individual psychotherapy (CAT) was found to have a similar cost-effectiveness profile when compared with each of the interventions providing the non-psychotherapy comparator (Bef). Similarly, Bef delivered within the specialised BPD service model (HYPE) was likely to be just as cost-effective as other alternatives, although it cost less and produced fewer health benefits when compared with YMHS + Bef. Negative incremental costs were likely driven by the lower occurrence of emergency department presentations and hospital admissions in the HYPE + Bef treatment arm (although the difference in presentations/admissions was not statistically significant).

Australian decision makers might be justified in adopting a much higher willingness-to-pay threshold than A$96,000 per QALY, given that personality disorder is the fourth leading cause of the mental health-related disease burden when compared to other mental disorders [27]; and is associated with high healthcare service use, dropout from education, unemployment, and engagement with the criminal justice and welfare systems [28–30]. At the nominated willingness-to-pay threshold, HYPE + CAT is the most likely intervention to be cost-effective with a 36.4% probability. This probability would increase with the adoption of a higher threshold.

There were no significant differences identified in the health outcome (i.e., QALYs) between the three treatment arms. Quality of life improved, with the utility weights approximately doubling across each arm. This could be attributable to a treatment effect, as cross-sectional data indicates that quality of life remains stable from adolescence to mid-adulthood among people with BPD [31]. This improvement is noteworthy as, upon entry into the trial, MOBY study participants reported severely impaired quality of life that was substantially worse than published norms among their same-age Australian peers (or Australians of any age), and also substantially worse than published findings among Australians with any other mental state or physical disorder, including cancer, cardiovascular disease, diabetes, and stroke [2, 32, 33]. Despite this improvement, by the end of the MOBY study follow-up period the mean utility weight (range: 0.536–0.617) was still below the mean utility weight of their same-aged Australian peers (0.828) [33]. Of course, the observed temporal improvements in quality of life could also be the result of regression toward the mean, rather than a treatment effect. The finding that no treatment arm was associated with a superior improvement in quality of life, when compared with one another, is consistent with the analyses of primary and secondary outcomes from the MOBY study [13].

With the exception of the HYPE + CAT versus HYPE + Bef comparison, this study found no significant differences in costs between the three treatment arms in terms of both the direct costs of delivering each intervention and all other healthcare costs associated with each arm of the trial over the 18-month follow-up period. This is despite higher rates of treatment attendance among study participants receiving individual psychotherapy and attending the specialist BPD service [13]. Emergency department presentations and hospital admissions were higher among those receiving HYPE + CAT and were a material contributor to the observed differences in total costs between each treatment arm (see Fig. 1). This might reflect greater opportunity in HYPE + CAT to detect crises and/or risks through more frequent contact and more in-depth discussions with young people, thereby increasing the likelihood that any response might involve an emergency department or inpatient admission. Psychiatric and general hospital admissions were rare, but very high-cost, events with considerable potential to inflate treatment costs through single instances. Nonetheless, these cost differences were not statistically significant and might simply be a chance finding. A longer follow-up period might also be required to adequately measure the occurrence of emergency department presentations and hospital admissions between treatment arms.

The results of this study are generally consistent with other economic evaluations of evidence-based treatments for BPD. A Norwegian within-trial economic evaluation conducted with 77 young people aged 12–18 years old (only 20.5% of whom were diagnosed with BPD) found no difference in overall costs or improvements in functioning between Dialectical Behaviour Therapy for Adolescents (DBT-A) and enhanced usual care (EUC). However, the study reported that DBT-A was significantly superior in reducing self-harm episodes [17]. The comparative cost-effectiveness analysis revealed that DBT-A was cost-effective compared with EUC for both these health outcomes (self-harm and functioning). However, QALYs were not measured in the study, and ‘functioning’ was measured using the Children’s Global Assessment Scale, a crude, single-item measure that conflates functioning and psychopathology, and has limited coverage of domains of functioning.

Finally, there are no studies comparing cost-effectiveness of *early* versus *late* intervention for BPD. Among the adult population with BPD, systematic reviews of within-trial cost-effectiveness evaluations have found unclear evidence regarding the cost-effectiveness of evidence-based treatments for BPD when compared with treatment as usual or other active comparators [14, 15]. These reviews have concluded that, while current data are lacking, treatments show promise for cost-effectiveness. The findings from cost-effectiveness evaluations to date are likely to be affected by small sample sizes, and variations in both methodology and statistical analyses that are associated with a high degree of uncertainty.

### Implications of findings

This economic evaluation has shown that, when using a willingness-to-pay threshold of A$96,000 per QALY, the specialist HYPE + CAT service model had a similar cost-effectiveness profile to the HYPE or generalist (YMHS) models combined with a non-psychotherapy (befriending) comparison. When examined in isolation, neither costs nor QALYs differed significantly between the treatment arms. Taken together with the clinical effectiveness findings, the results support the delivery of structured early intervention programs for BPD in specialist and primary youth mental health settings, such as HYPE and *headspace*. The essential characteristics of these programs are outlined elsewhere [34, 35]. These programs are not reliant upon individual psychotherapy to be clinically effective or good value for money. However, it is noteworthy that the clinical needs of four YMHS + Bef participants exceeded the capacity of the available *headspace* clinical resources, requiring discontinuation from the trial intervention and referral to Orygen/HYPE for specialised care. YMHS + Bef was designed specifically for MOBY and is difficult to implement within the current *headspace* funding model until it has capacity for salaried (not fee-for-service) staff and outreach care when necessary. Further research is required to better match interventions to individuals’ needs, including the indications for, and timing of, individual psychotherapy within these programs.

### Limitations

This study has several limitations. First, the primary comparator used in this study (YMHS + Bef) represents a comparison condition designed specifically for the MOBY trial, that is not representative of usual *headspace* care for young people with BPD. It is consequently not possible to determine whether the specialist HYPE mental health service is cost-effective with respect to usual care. Second, small sample sizes might mean that this study is insufficiently powered to reliably detect small-to-medium differences in costs and QALYs between treatment arms, bearing in mind that the trial was powered to detect differences in the joint primary outcomes, not secondary (cost-effectiveness) outcomes. The statistical power of the study was further reduced by missing AQoL-8D utility data at follow-up. Third, treatment data might underestimate true service usage, as they were partly collected through researcher interviews. In addition, once a participant was discharged from *headspace*/Orygen, data on emergency department presentations and inpatient admissions were not collected. Fourth, using the healthcare sector perspective excludes non-health outcomes and costs or savings that might arise from early intervention (e.g., effects upon education, employment, housing and involvement with the justice system), which have been demonstrated to be substantial in BPD [36–38]. Similarly, the cost of informal care provided by family and other caregivers was not considered. Fifth, the 18-month time horizon might be too short to detect long-term changes in health and other non-health outcomes over time. Sixth, the cost analysis directly quantified variable costs around the time spent by staff delivering each of the three interventions. Economies of scale due to the fixed costs of employing salaried staff at *headspace*/Orygen were thus not accounted for. Seventh, the high degree of missing data observed across both the cost and utility weight data meant that the results of the multiple imputation analysis might be susceptible to bias, particularly if missing data were a consequence of being ‘missing not at random’.

## Conclusion

Providing early intervention for young people through a comprehensive specialist treatment (HYPE+CAT) was likely to be just as cost-effective as a generalist service delivered through an enhanced primary care platform (YMHS+Bef) when adopting a previously cited willingness-to-pay threshold of A$96,000 per QALY. Likewise, the specialist mental health service model without individual therapy (HYPE+Bef) had similar cost-effectiveness credentials. However, observed differences between the three treatment arms might not have reached statistical significance due to high levels of uncertainty around incremental costs and health outcomes. Taken together with the clinical effectiveness findings [13], these findings provide further support for the delivery of structured early intervention for BPD programs in both generalist, enhanced primary care settings, and in specialist tertiary mental health services.

## Supplementary Information

Below is the link to the electronic supplementary material.

Supplementary Material 1

## Data Availability

Access to data and materials can be requested via the Health Data Australia catalogue (http://researchdata.edu.au/health).
