# Supplementary material for: Economic evaluation of three forms of early intervention for young people with borderline personality disorder: a within-trial cost-utility analysis from the MOBY clinical trial
Source: Eur Child Adolesc Psychiatry. 2026 Mar 16;35(6):1935–46. doi: 10.1007/s00787-026-02994-9 (PMC13337604; doi:10.1007/s00787-026-02994-9)
Supplement: Supplementary file 1 — Supplementary Material 1 [file 787_2026_2994_MOESM1_ESM.pdf]

Supplementary appendices

Appendix S1 – Cost analysis methods 2

Appendix S2 – Health economics checklists 3

Overview 3

Appendix S3 – Analysis of missing data mechanisms 8

Overview 8

Analysis of missing data patterns 9

Logistic regression analyses 13

Conclusion 17

Appendix S4 – Sample characteristics at baseline 18

Overview 18

Appendix S5 – Unit cost data 20

Appendix S6 – Summary of cost and utility weight data 23

References 25

Appendix S1 – Cost analysis methods

Appendix Table 1 Description of methods used to derive each of the four cost types

Cost type	Description
1. Direct MOBY intervention cost	The direct cost of delivering each of the three MOBY interventions comprised the cost of staff members delivering: (1) CAT/Bef sessions to study participants; and (2) other intervention sessions provided by the HYPE/YMHS service models (e.g., case management, psychiatric medical reviews, family sessions, vocational support for work/study, psychosocial recovery program, etc). Records maintained during the MOBY trial were used to determine the amount of time each staff member spent delivering CAT/Bef and other intervention sessions to participants. Data were obtained from the Australian Bureau of Statistics (ABS) Employee, Earnings and Hours survey on the average hourly wage for each staff member, based on their professional qualification.[1] All wage rates included a 30% loading to account for on-costs (e.g., annual leave, sick leave, superannuation, etc) and overheads (e.g., infrastructure and equipment provided by Orygen and *headspace*). Overheads were assumed to be similar across services provided at the trial sites. The MOBY interventions ended after 16 CAT/Bef sessions had been offered/attended or when a participant did not attend a CAT/Bef session after six consecutive weeks. Treatment completion was defined as attending ≥8 CAT/Bef sessions.[2] The costs associated with training staff members to deliver intervention sessions were excluded from the analysis as they are sunk costs – i.e., costs that have already been incurred, are unrecoverable and do not factor into future decision making.
2. Cost of other healthcare services	The cost of other healthcare services not related to the MOBY intervention comprised: (1) any additional intervention sessions accessed from HYPE/YMHS after the MOBY intervention ended; and (2) any visits to a healthcare professional to support mental health – e.g., general practitioner (GP), psychologist, counsellor, etc. Records maintained during the MOBY trial were used to track the number and duration of HYPE/YMHS intervention sessions. Additionally, a resource use questionnaire was developed by the research team to record any healthcare professional visits (and psychotropic medication use) reported by participants during the MOBY trial. This was administered at baseline and follow-up at 3, 6, 12 and 18 months. Unit costs for GP visits were obtained from the Medicare Benefits Schedule;[3] while unit costs for all other healthcare professional visits were based on hourly wages from the ABS Employee, Earnings and Hours survey (inclusive of 30% loading).[1]
3. Cost of psychotropic medications	The cost of psychotropic medications was estimated by using participant-reported data on the duration and maximum dose of each psychotropic drug taken during the MOBY trial. It was conservatively assumed that the maximum dose of each drug (in milligrams) would apply across the entire time period during which medication use was reported. Pharmaceutical Benefits Scheme data were used to derive unit costs for psychotropic medications.[4]
4. Cost of emergency department presentations and hospital admissions	The cost of emergency department presentations and hospital admissions comprised all psychiatric inpatient admissions, general inpatient admissions and emergency department presentations that occurred during the episode of care received after randomisation. Data on the total length of stay for each hospital admission were collated using administrative data routinely collected as part of clinical care at Orygen/*headspace*. Unit costs for inpatient admissions and emergency department presentations were obtained from the Independent Health and Aged Care Pricing Authority.[5]

Appendix S2 – Health economics checklists

Overview

This appendix presents two health economics checklists that were adhered to by the current economic evaluation study. The first is the Consolidated Health Economic Evaluation Reporting Standards (CHEERS) statement (see Appendix Table 2).[6] The second is the impact inventory recommended by the Second Panel on Cost-Effectiveness in Health and Medicine (see Appendix Table 3).[7]

Appendix Table 2 Consolidated Health Economic Evaluation Reporting Standards (CHEERS) statement

Section/item	Item No	Recommendation	Section reported
Title and abstract			
Title	1	Identify the study as an economic evaluation or use more specific terms such as "cost-effectiveness analysis", and describe the interventions compared.	See Title
Abstract	2	Provide a structured summary of objectives, perspective, setting, methods (including study design and inputs), results (including base case and uncertainty analyses), and conclusions.	See Abstract
Introduction			
Background and objectives	3	Provide an explicit statement of the broader context for the study.	See Introduction
		Present the study question and its relevance for health policy or practice decisions.	See Introduction
Methods			
Target population and subgroups	4	Describe characteristics of the base case population and subgroups analysed, including why they were chosen.	See 'Intervention trial' subsection in the Methods
Setting and location	5	State relevant aspects of the system(s) in which the decision(s) need(s) to be made.	See 'Intervention trial' subsection in the Methods
Study perspective	6	Describe the perspective of the study and relate this to the costs being evaluated.	See 'Cost analysis' and 'Statistical analysis' subsections in the Methods

Section/item	Item No	Recommendation	Section reported
Comparators	7	Describe the interventions or strategies being compared and state why they were chosen.	See 'Statistical analysis' subsection in the Methods
Time horizon	8	State the time horizon(s) over which costs and consequences are being evaluated and say why appropriate.	See 'Cost analysis' & 'Health outcomes' subsections in the Methods
Discount rate	9	Report the choice of discount rate(s) used for costs and outcomes and say why appropriate.	See 'Cost analysis' & 'Statistical analysis' subsections in the Methods
Choice of health outcomes	10	Describe what outcomes were used as the measure(s) of benefit in the evaluation and their relevance for the type of analysis performed.	See 'Health outcomes' subsection in the Methods
Measurement of effectiveness	11a	Single study-based estimates: Describe fully the design features of the single effectiveness study and why the single study was a sufficient source of clinical effectiveness data.	See 'Health outcomes' subsection in the Methods
	11b	Synthesis-based estimates: Describe fully the methods used for identification of included studies and synthesis of clinical effectiveness data.	Not applicable
Measurement and valuation of preference-based outcomes	12	If applicable, describe the population and methods used to elicit preferences for outcomes.	See 'Health outcomes' subsection in the Methods
Estimating resources and costs	13a	Single study-based economic evaluation: Describe approaches used to estimate resource use associated with the alternative interventions. Describe primary or secondary research methods for valuing each resource item in terms of its unit cost. Describe any adjustments made to approximate to opportunity costs.	See 'Cost analysis' subsection in the Methods
	13b	Model-based economic evaluation: Describe approaches and data sources used to estimate resource use associated with model health states. Describe primary or secondary research methods for valuing each resource item in terms of its unit cost. Describe any adjustments made to approximate to opportunity costs.	Not applicable
Currency, price date, and conversion	14	Report the dates of the estimated resource quantities and unit costs. Describe methods for adjusting estimated unit costs to the year of reported costs if necessary. Describe methods for converting costs into a common currency base and the exchange rate.	See 'Cost analysis' subsection in the Methods
Choice of model	15	Describe and give reasons for the specific type of decision-analytical model used. Providing a figure to show model structure is strongly recommended.	Not applicable
Assumptions	16	Describe all structural or other assumptions underpinning the decision-analytical model.	Not applicable

Section/item	Item No	Recommendation	Section reported
Results			
Study parameters	18	Report the values, ranges, references, and, if used, probability distributions for all parameters. Report reasons or sources for distributions used to represent uncertainty where appropriate. Providing a table to show the input values is strongly recommended.	See Results in the main manuscript; as well as Appendices S3 to S4.
Incremental costs and outcomes	19	For each intervention, report mean values for the main categories of estimated costs and outcomes of interest, as well as mean differences between the comparator groups. If applicable, report incremental cost-effectiveness ratios.	See Results and Table 3 in the main manuscript.
Characterising uncertainty	20a	Single study-based economic evaluation: Describe the effects of sampling uncertainty for the estimated incremental cost and incremental effectiveness parameters, together with the impact of methodological assumptions (such as discount rate, study perspective).	See Results, Table 3 and Figure 3 in the main manuscript.
	20b	Model-based economic evaluation: Describe the effects on the results of uncertainty for all input parameters, and uncertainty related to the structure of the model and assumptions.	Not applicable
Characterising heterogeneity	21	If applicable, report differences in costs, outcomes, or cost-effectiveness that can be explained by variations between subgroups of patients with different baseline characteristics or other observed variability in effects that are not reducible by more information.	See Results
Discussion			
Study findings, limitations, generalisability, and current knowledge	22	Summarise key study findings and describe how they support the conclusions reached. Discuss limitations and the generalisability of the findings and how the findings fit with current knowledge.	See Discussion
Other			
Source of funding	23	Describe how the study was funded and the role of the funder in the identification, design, conduct, and reporting of the analysis. Describe other non-monetary sources of support.	See Funding
Conflicts of interest	24	Describe any potential for conflict of interest of study contributors in accordance with journal policy. In the absence of a journal policy, we recommend authors comply with International Committee of Medical Journal Editors recommendations.	See Declaration of Conflicting Interests

Appendix Table 3 Impact inventory as recommended by the Second Panel on Cost-Effectiveness in Health and Medicine

Sector	Type of impact	Included in this reference case analysis from … perspective?		Notes on sources of evidence
		Health sector	Societal	
**Formal Health Care Sector**				
Health	*Health outcomes (effects)*			
	Longevity effects	N/A	N/A	
	Health-related quality-of-life effects	✓	N/A	AQoL-8D
	Other health effects (e.g., adverse events and secondary transmissions of infections)	N/A	N/A	
	Medical costs			
	Paid for by third-party payers	✓	N/A	Medications, consultations, hospital care reimbursed by government
	Paid for by patients out-of-pocket	✓	N/A	Gap fees for medications, consultations, hospital care
	Future related medical costs (payers and patients)	N/A	N/A	
	Future unrelated medical costs (payers and patients)	N/A	N/A	
**Informal Health Care Sector**				
Health	Patient-time costs	N/A	N/A	
	Unpaid caregiver-time costs	N/A	N/A	
	Transportation costs	N/A	N/A	

Sector	Type of impact	Included in this reference case analysis from … perspective?		Notes on sources of evidence
		Health sector	Societal	
**Non-Health Care Sectors (with examples of possible items)**				
Productivity	Labour market earnings lost	N/A	N/A	
	Cost of unpaid lost productivity due to illness	N/A	N/A	
	Cost of uncompensated household production	N/A	N/A	
Consumption	Future consumption unrelated to health	N/A	N/A	
Social Services	Cost of social services as part of intervention	N/A	N/A	
**Non-Health Care Sectors (with examples of possible items)**				
Legal or criminal justice	Number of crimes related to intervention	N/A	N/A	
	Cost of crimes related to intervention	N/A	N/A	
Education	Impact of intervention on educational achievement of population	N/A	N/A	
Housing	Cost of intervention on home improvements (e.g., removing lead paint)	N/A	N/A	
Environment	Production of toxic waste or pollution by intervention	N/A	N/A	
Other (specify)		N/A	N/A	

Table adapted from: Sanders et al.[7]

Appendix S3 – Analysis of missing data mechanisms

Overview

The purpose of this section is to investigate the underlying mechanisms for missing data across the cost and utility weight variables. These mechanisms involve data that are: (1) missing completely at random [MCAR]; (2) missing at random [MAR]; and (3) missing not at random [MNAR]. Data are considered 'missing completely at random' when missing responses do not depend on either the observed data or the value of the missing variables. By contrast, data are considered 'missing at random' when missing responses are systematically related to other observed variables (e.g., when missing responses are associated with baseline demographic variables such as age or gender). Lastly, data are considered 'missing not at random' if there is a systematic relationship between the value of the missing variable and the likelihood of a missing response (e.g., when participants who earn a high level of income are less likely to provide responses to variables asking them to self-report their individual income level).[8] The use of multiple imputation methods as a means of addressing missing data are valid when the underlying missing data mechanism is either 'missing completely at random' or 'missing at random'.[8] If data were 'missing not at random', then this would lead to biased statistical inferences. The mechanisms underlying missing responses for utility weights (i.e., health outcomes) and cost variables were consequently analysed by: (1) investigating overall patterns of missing data; and (2) performing multivariate logistic regression analyses to determine if data missingness was related to other observed variables collected at baseline. The focus of these analyses was to explore whether the mechanisms for missing cost and utility weight data were, at a minimum, due to data being 'missing at random'.

Analysis of missing data patterns

Appendix Table 4 presents the frequency of study participants with complete cost data, utility weight data and primary outcome data, by follow-up. Cost data involving the 'Direct MOBY intervention cost' and the 'Cost of emergency department presentations and hospital admissions' have not been presented in this table as they were collected using administrative clinical records; and were thus considered complete data over the entire follow-up period of 18 months. By contrast, cost data on the 'Cost of other healthcare services' and the 'Cost of psychotropic medications' were collected from participants using a resource use questionnaire administered at each follow-up period – i.e., baseline, 3 months, 6 months 12 months and 18 months. Participants who were unable to provide responses at a particular follow-up period were asked to recall their responses for missed follow-up periods at later a later follow-up, if possible. It was observed that the likelihood of missing cost data progressively increased at each subsequent follow-up period. This led to a total of 70 participants (50.4%) having complete data on total costs at 18 months. Similarly, the likelihood of missing utility weight data was observed to progressively increase at each subsequent follow-up period. This led to a total of 59 participants (42.4%) having complete data on total QALYs at 18 months. Overall, a total of 44 participants (31.6%) had complete data on both total costs and total QALYS at 18 months.

Patterns of missing data on the primary outcome generally tracked patterns of missing data for the cost and utility weight data across each follow-up period. Though marginally higher proportions of missing data were observed for cost and utility weight data when compared to primary outcome data. This observation can be explained by the fact that, during follow-up interviews, greater priority was given to the collection of data on the primary outcome in place of secondary outcomes (i.e., costs and utility weight data). As such, a greater proportion of cost and utility weight data were missing each follow-up periods when compared to the primary outcome – e.g., at the 18-month follow-up period, a total of: 89 participants (64.0%) had complete primary outcome data; 86 participants (61.9%) had complete utility weight data; and 70 participants (50.4%) had complete data on the 'Cost of other healthcare services' and the 'Cost of psychotropic medications'.

Appendix Table 4 Summary of study participants with complete data on AQoL-8D utility weights and costs

Data type (by follow-up period)	HYPE+CAT (n = 46)	HYPE+Bef (n = 46)	YMHS+Bef (n = 47)
Data on the cost of other healthcare services and the cost of psychotropic medications, n (%)			
Baseline	46 (100.0%)	46 (100.0%)	46 (97.8%)
3-month follow-up	34 (73.9%)	39 (84.8%)	35 (74.5%)
6-month follow-up	34 (73.9%)	33 (71.7%)	35 (74.5%)
12-month follow-up	28 (60.9%)	29 (63.0%)	34 (72.3%)
18-month follow-up	20 (43.5%)	26 (56.5%)	24 (51.0%)
Data on total costs [a], n (%)			
18-month follow-up	20 (43.5%)	26 (56.5%)	24 (51.0%)
Utility weight data, n (%)			
Baseline	46 (100.0%)	46 (100.0%)	47 (100.0%)
3-month follow-up	31 (67.4%)	40 (87.0%)	36 (76.6%)
6-month follow-up	32 (69.6%)	30 (65.2%)	34 (72.3%)
12-month follow-up	26 (56.5%)	32 (69.6%)	33 (70.2%)
18-month follow-up	22 (47.8%)	35 (76.1%)	29 (61.7%)
Data on total QALYs [b], n (%)			
18-month follow-up	14 (30.4%)	23 (50.0%)	22 (46.8%)
Primary outcome data [c], n (%)			
3-month follow-up	34 (73.9%)	41 (89.1%)	41 (87.2%)
6-month follow-up	34 (73.9%)	33 (71.7%)	35 (74.5%)
12-month follow-up	28 (60.9%)	35 (76.1%)	35 (74.5%)
18-month follow-up	24 (52.2%)	36 (78.3%)	29 (61.7%)

Abbreviations: AQoL-8D - Assessment of Quality of Life-8D; Bef - befriending; CAT - cognitive analytic therapy; HYPE - Helping Young People Early; QALYs - quality-adjusted life years; YMHS - Youth Mental Health Service.

[a] This comprises all resource use data collected in relation to MOBY intervention sessions received across each treatment arm, as well as other healthcare resources (i.e., other healthcare services not related to the MOBY intervention, ED presentations & hospital admissions and psychotropic medications). If a participant had missing cost values at one or more follow-up periods, then the total cost estimate would also be missing.

[b] Total QALYs were estimated by using area-under-the-curve methods to convert utility weights observed at each follow-up period into a corresponding QALY estimate for each participant. If a participant had missing utility weights at one or more follow-up periods, then the total QALY estimate would also be missing.

[c] The primary outcome was psychosocial functioning, jointly measured by the Inventory of Interpersonal Problems Circumplex Version and the Social Adjustment Scale Self-report.[2]

Appendix Table 5 provides a summary of missing data patterns across participants in relation to the cost and utility weight data collected at each follow-up period. Complete data on costs and utility weights were available for ~32% of respondents. Missing data are considered monotone when: (1) a missing value is observed and all values to the right of its position are also missing; and (b) a non-missing value is observed and all values to the left of its position are also non-missing.[8] Monotone missing data patterns are typically caused by loss to follow up. Overall, missing utility weight data were observed to be monotone for ~43% of respondents. Non-monotone missing data patterns were observed across the remaining ~25% of respondents.

Appendix Table 6 provides a summary of missing data patterns across participants for aggregate cost and QALY data at 18 months – i.e., the 'Cost of other healthcare services' and the 'Cost of psychotropic medications', as well as total costs and total QALYs at 18 months. As before, complete data were available for ~32% of respondents. Data were completely missing for aggregate cost and QALY data across ~39% of participants. By contrast: ~19% of participants had complete cost data but missing QALY data; while ~10% of participants had complete QALY data but missing cost data. It should be noted that, when estimating total costs/QALYs for a given participant over the entire 18-month follow-up period, a single missing value for cost/utility data at a particular follow-up period ultimately results in the omission of all remaining, non-missing cost/utility data observed for that participant across the remaining follow-up periods. For example, when estimating total costs, a single missing value for the 'Cost of other healthcare services' or the 'Cost of psychotropic medications' can lead to the omission of complete data on 'Direct MOBY intervention cost' and the 'Cost of emergency department presentations and hospital admissions'.

Appendix Table 5 Summary of missing data patterns across participants for cost and utility weight data that is collected over time

	Missing-value patterns (1 means complete)													
Percent	CostOTHR - Baseline	CostOTHR - 3 months	CostOTHR - 6 months	CostOTHR - 12 months	CostOTHR - 18 months	CostMEDS - Baseline	CostMEDS - 3 months	CostMEDS - 6 months	CostMEDS - 12 months	CostMEDS - 18 months	Utility - 3 months	Utility - 6 months	Utility - 12 months	Utility - 18 months
32%	1	1	1	1	1	1	1	1	1	1	1	1	1	1
7%	1	0	0	0	0	1	0	0	0	0	0	0	0	0
7%	1	1	1	1	0	1	1	1	1	0	1	1	1	1
4%	1	1	1	1	1	1	1	1	1	1	1	1	1	0
4%	1	1	1	1	1	1	1	1	1	1	1	0	0	1
3%	1	0	0	0	0	1	0	0	0	0	1	1	1	0
3%	1	1	1	1	1	1	1	1	1	1	1	0	1	1
3%	1	1	1	1	1	1	1	1	1	1	1	1	0	1
2%	1	1	0	0	0	1	1	0	0	0	0	0	0	0
2%	1	1	1	0	0	1	1	1	0	0	1	1	0	0
2%	1	1	1	0	0	1	1	1	0	0	1	1	1	0
2%	1	1	1	1	0	1	1	1	1	0	1	1	1	0
1%	1	0	0	0	0	1	0	0	0	0	0	0	0	1
1%	1	0	0	0	0	1	0	0	0	0	0	0	1	0
1%	1	0	0	0	0	1	0	0	0	0	1	0	0	0
1%	1	0	0	0	0	1	0	0	0	0	1	1	1	1
1%	1	1	1	1	0	1	1	1	1	0	0	1	1	1
1%	1	1	1	1	1	1	1	1	1	1	0	1	1	1
1%	1	1	1	1	0	1	1	1	1	0	1	0	0	0
1%	1	1	1	0	0	1	1	1	0	0	1	1	1	1
<1%	0	0	0	0	0	0	0	0	0	0	0	0	0	0
<1%	1	0	0	0	0	1	0	0	0	0	0	1	0	0
<1%	1	0	0	0	0	1	0	0	0	0	0	1	1	0
<1%	1	0	0	0	0	1	0	0	0	0	1	0	0	1
<1%	1	0	0	0	0	1	0	0	0	0	1	0	1	1
<1%	1	0	0	0	0	1	0	0	0	0	1	1	0	1
<1%	1	1	1	0	0	1	1	1	0	0	0	0	0	0
<1%	1	1	1	1	0	1	1	1	1	0	0	0	0	0
<1%	1	1	1	1	1	1	1	1	1	1	0	0	0	0
<1%	1	1	1	1	1	1	1	1	1	1	0	0	0	1
<1%	1	1	1	1	0	1	1	1	1	0	0	0	1	0
<1%	1	1	1	1	1	1	1	1	1	1	0	0	1	0
<1%	1	1	1	1	1	1	1	1	1	1	0	1	0	1
<1%	1	1	1	0	0	1	1	1	0	0	0	1	1	0
<1%	1	1	0	0	0	1	1	0	0	0	1	0	0	0
<1%	1	1	0	0	0	1	1	0	0	0	1	0	1	0
<1%	1	1	0	0	0	1	1	0	0	0	1	1	0	0
<1%	1	1	0	0	0	1	1	0	0	0	1	1	0	1
<1%	1	1	0	0	0	1	1	0	0	0	1	1	1	1
<1%	1	1	1	0	0	1	1	1	0	0	1	0	0	0
<1%	1	1	1	1	0	1	1	1	1	0	1	0	0	1
<1%	1	1	1	0	0	1	1	1	0	0	1	1	0	1
<1%	1	1	1	1	0	1	1	1	1	0	1	1	0	0
<1%	1	1	1	1	1	1	1	1	1	1	1	1	0	0

Abbreviations: CostOTHR - Cost of other healthcare services; CostMEDS - Cost of psychotropic medications.

Note: Rows highlighted in yellow involve a monotone pattern of missing data, such that: (1) when a value is missing, all values to the right of its position are also missing; and (b) when a non-missing value is observed, all values to the left of this value are also non-missing.

Appendix Table 6 Summary of missing data patterns across participants for aggregate cost and QALY data

	**Missing-value patterns** (1 means complete)			
**Percent**	**CostOTHR - Total**	**CostMEDS - Total**	**Total Costs**	**Total QALYs**
32%	1	1	1	1
39%	0	0	0	0
19%	1	1	1	0
10%	0	0	0	1

Abbreviations: CostOTHR - Cost of other healthcare services; CostMEDS - Cost of psychotropic medications.

Logistic regression analyses

A variable is considered to have values that are 'missing at random' when missing responses are systematically related to other observed variables within the dataset. A multivariate logistic regression was performed to analyse whether missing responses to the cost variables and the utility weight variables were associated with any of the baseline demographic variables. These included variables comprising: the treatment arm; age; gender; sociodemographic disadvantage; being born in Australia; living with a biological parent; not being in a relationship; achieving age-appropriate secondary-school milestones; the number of current psychiatric diagnoses; not being in education, employment, training or homemaking; and the Social and Occupational Functioning Assessment Scale (SOFAS).

The results of the logistic regression for the missing cost variables are presented in Appendix Table 7. Overall, no significant associations were observed between baseline demographic variables and missing cost data.

The results of the logistic regression for missing utility variables are presented in Appendix Table 8. Two variables involving the treatment arm and the Social and Occupational Functioning Assessment Scale (SOFAS) were significantly associated with missing utility responses. It follows that there is evidence to suggest that missing utility weight data are 'missing at random', given that missing utility weight values were found to be associated with these two baseline demographic variables. Moreover, there is no *a priori* reason to believe that there is a systematic relationship underlying participant non-response, such that utility weight data were 'missing not at random'. For example, an analysis of missing data patterns among the eight dimensions that compose the aggregate AQoL-8D utility scores at each follow-up period (i.e., independent living, senses, pain, mental health, happiness, self-worth, coping and relationships) revealed that dimension values were uniformly missing across all eight dimensions for every participant and at every follow-up period. This suggests that missing data was the result of loss to follow-up. If missing utility weight data were 'missing not at random' then a greater frequency of missing values might be observed for a narrow set of dimensions, rather than uniformly missing values across all eight dimensions. Nevertheless, an alternative explanation could be that loss to follow-up could be the result of adverse outcomes related to the primary outcome and, by proxy, health-related quality of life and the resulting utility weights.

Appendix Table 7 Results of the multivariate logistic regression model analysing the relationship between missing cost values and baseline demographic variables

Covariate	Odds ratio	95% CI	p-value
Treatment arm	0.88	(0.57 to 1.35)	0.553
Age	0.94	(0.80 to 1.10)	0.428
Gender=Male (base level)	1.00		
Gender=Female	0.99	(0.39 to 2.48)	0.979
Sociodemographic disadvantage=Low (base level)	1.00		
Sociodemographic disadvantage=Medium	0.68	(0.26 to 1.82)	0.446
Sociodemographic disadvantage=High	0.49	(0.17 to 1.40)	0.182
Born in Australia=Australian born (base level)	1.00		
Born in Australia=Other	1.08	(0.36 to 3.28)	0.887
Living with a biological parent=No (base level)	1.00		
Living with a biological parent=Yes	1.03	(0.47 to 2.27)	0.943
Relationship status=No relationship (base level)	1.00		
Relationship status=In a relationship/married	0.99	(0.47 to 2.06)	0.973
Achieved age-appropriate secondary-school milestones=no (base level)	1.00		
Achieved age-appropriate secondary-school milestones=yes	0.46	(0.19 to 1.12)	0.088
Number of current psychiatric diagnoses at baseline	1.07	(0.83 to 1.37)	0.617
Not in education, employment, training or homemaking=No (base level)	1.00		
Not in education, employment, training or homemaking=Yes	0.54	(0.25 to 1.20)	0.129
Social and Occupational Functioning Assessment Scale (SOFAS)	0.97	(0.92 to 1.01)	0.165
Intercept	84.66	(0.60 to11,963)	0.079

Abbreviations: 95% CI - 95% confidence interval.

** Statistically significant change at the 5% level of significance (i.e., p < 0.05).*

Appendix Table 8 Results of the multivariate logistic regression model analysing the relationship between missing utility weight values and baseline demographic variables

Covariate	Odds ratio	95% CI	p-value
Treatment arm	0.63	(0.39 to 0.99)	0.049 *
Age	1.17	(0.98 to 1.40)	0.079
Gender=Male (base level)	1.00		
Gender=Female	1.72	(0.66 to 4.49)	0.270
Sociodemographic disadvantage=Low (base level)	1.00		
Sociodemographic disadvantage=Medium	0.47	(0.15 to 1.44)	0.187
Sociodemographic disadvantage=High	0.50	(0.15 to 1.63)	0.250
Born in Australia=Australian born (base level)	1.00		
Born in Australia=Other	0.38	(0.12 to 1.27)	0.116
Living with a biological parent=No (base level)	1.00		
Living with a biological parent=Yes	0.59	(0.25 to 1.37)	0.218
Relationship status=No relationship (base level)	1.00		
Relationship status=In a relationship/married	1.24	(0.57 to 2.69)	0.584
Achieved age-appropriate secondary-school milestones=no (base level)	1.00		
Achieved age-appropriate secondary-school milestones=yes	1.66	(0.64 to 4.29)	0.293
Number of current psychiatric diagnoses at baseline	0.93	(0.71 to 1.22)	0.614
Not in education, employment, training or homemaking=No (base level)	1.00		
Not in education, employment, training or homemaking=Yes	0.93	(0.41 to 2.12)	0.867
Social and Occupational Functioning Assessment Scale (SOFAS)	0.95	(0.90 to 0.99)	0.049 *
Intercept	5.46	(0.03,1,022)	0.525

Abbreviations: 95% CI - 95% confidence interval.

** Statistically significant change at the 5% level of significance (i.e., p < 0.05).*

Conclusion

Overall, it appears that missing cost and utility weight data were the result of a combination of: (1) loss to follow-up; and (2) missing values resulting from the priority given to the collection of primary outcome data (i.e., psychosocial functioning) over and above secondary outcomes (i.e., cost and utility weight data). There is a possibility that data is, at a minimum, 'missing at random'. However, the possibility also remains that data is 'missing not at random' if the missing values associated with the primary outcome, cost or utility variables are causally related to loss to follow-up and/or non-responses. For the purposes of the statistical analysis, we had opted to proceed with multiple imputation on the assumption that data is 'missing at random'. This decision was made to leverage available data on costs and utility weights that would otherwise be omitted when analysing complete case data on total costs and total QALYs. That is, when estimating total costs/QALYs for a given participant across the entire 18-month follow-up period, a single missing value for cost/utility data at a particular follow-up period will lead to the omission of all remaining non-missing cost/utility data observed at other follow-up periods. In choosing to proceed with multiple imputation, there is a risk that the results of the analysis are biased due to the high degree of missing data (>40%) or the possibility of data being 'missing not at random'.[8] A sensitivity analysis was thus performed to explore the impact of limiting the analysis to complete cases only – i.e., the 44 participants (31.6%) who had complete data on both total costs and total QALYS at 18 months.

Appendix S4 – Sample characteristics at baseline

Overview

Baseline demographic and clinical variables are presented in Appendix Table 9 for the total sample and across the three treatment arms.

Appendix Table 9 Baseline demographic and clinical characteristics by treatment arms

Characteristic	HYPE+CAT (n = 46)	HYPE+Bef (n = 46)	YMHS+Bef (n = 47)	Total sample (N = 139)
Age, mean (SD)	19.0 (2.9)	19.1 (2.6)	19.0 (2.8)	19.1 (2.8)
Female, n (%)	37 (80.4)	36 (78.3)	39 (83.0)	112 (80.6)
Sociodemographic disadvantage [a], n (%)				
Low	6 (13.0)	11 (23.9)	8 (17.0)	25 (18.0)
Medium	26 (56.5)	21 (45.7)	22 (46.8)	69 (49.6)
High	14 (30.4)	14 (30.4)	17 (36.2)	45 (32.4)
Born in Australia, n (%)	38 (82.6)	40 (87.0)	45 (95.7)	123 (88.5)
Living with a biological parent, n (%)	26 (56.5)	32 (69.6)	26 (55.3)	84 (60.4)
Not in a relationship, n (%)	27 (58.7)	23 (50.0)	32 (68.1)	82 (59.0)
Age-appropriate secondary-school milestones [b], n (%)	30 (65.2)	29 (63.0)	32 (68.1)	91 (65.6)
Number of current psychiatric diagnoses, mean (SD)	2.6 (1.6)	2.5 (1.5)	2.6 (1.5)	2.6 (1.5)
Number of current personality disorder diagnoses [c], mean (SD)	2.6 (1.5)	2.0 (1.2)	2.4 (1.5)	2.4 (1.4)
Not in education, employment, training, or homemaking, n (%)	18 (39.1)	19 (41.3)	19 (40.4)	56 (40.2)
SOFAS, mean (SD)	57.8 (7.3)	58.4 (8.8)	58.1 (7.9)	58.1 (0.5)

Abbreviations: Bef - befriending; BPD - borderline personality disorder; CAT - cognitive analytic therapy; HYPE - Helping Young People Early; SD - standard deviation; SOFAS - Social and Occupational Functioning Assessment Scale; YMHS - Youth Mental Health Service.

[a] *Index of Relative Social Disadvantage, Australian Bureau of Statistics.*

[b] *Age-appropriate milestones comprise: Year 12 if 19 years or older; Year 11 if 18 years old; Year 10 if 17 years old; Year 9 if 16 years old; and Year 8 or below if 15 years old.*

[c] *Includes borderline personality disorder.*

Appendix S5 – Unit cost data

Appendix Table 10 Unit cost data for healthcare services

Cost category	Healthcare service	Description	Unit cost (2015 A$)	Source
MOBY intervention – CAT/Bef sessions	CAT	50-minute individual session provided by a psychologist	46.29	ABS [1]
	Befriending	50-minute individual session provided by a psychologist	46.29	ABS [1]
MOBY intervention – Other intervention sessions	Case management	50-minute individual session provided by a psychologist	46.29	ABS [1]
	Psychiatric medical review	50-minute individual session provided by a psychiatrist	98.24	ABS [1]
	Family work (individual)	50-minute individual session provided by a psychologist	46.29	ABS [1]
	Family work (group)	120-minute group session provided by two psychologists	27.77	ABS [1]
	General practitioner	Average MBS fee for item codes 3, 23, 36 and 44	42.33	MBS [3]
	Psychologist	50-minute individual session provided by a psychologist	46.29	ABS [1]
	Specialist vocational (employment/education) support	50-minute individual session provided by a vocational specialist	46.29	ABS [1]
	Vocational (education) support	120-minute group session provided by a psychologist	13.89	ABS [1]
	Psychosocial recovery program	90-minute group session provided by two psychologists	20.83	ABS [1]
	Neuropsychology assessment	90-minute individual session provided by a neuropsychologist	83.32	ABS [1]
Other healthcare services – Other intervention sessions	Case management	50-minute individual session provided by a psychologist	46.29	ABS [1]
	Psychiatric medical review	50-minute individual session provided by a psychiatrist	98.24	ABS [1]
	Family work (individual)	50-minute individual session provided by a psychologist	46.29	ABS [1]
	Family work (group)	120-minute group session provided by two psychologists	27.77	ABS [1]

Cost category	Healthcare service	Description	Unit cost (2015 A$)	Source
	General practitioner	Average MBS fee for item codes 3, 23, 36 and 44	42.33	MBS [3]
	Psychologist	50-minute individual session provided by a psychologist	46.29	ABS [1]
	Specialist vocational (employment/education) support	50-minute individual session provided by a vocational specialist	46.29	ABS [1]
	Vocational (education) support	120-minute group session provided by a psychologist	13.89	ABS [1]
	Psychosocial recovery program	90-minute group session provided by two psychologists	20.83	ABS [1]
	Neuropsychology assessment	90-minute individual session provided by a neuropsychologist	83.32	ABS [1]
Other healthcare services – Healthcare professional visits	General practitioner	Average MBS fee for item codes 3, 23, 36 and 44	42.33	MBS [3]
	Psychologist	50-minute individual session provided by a psychologist	46.29	ABS [1]
	Psychiatrist	50-minute individual session provided by a psychiatrist	98.24	ABS [1]
	Social worker	50-minute individual session provided by a social worker	36.55	ABS [1]
	Counsellor / School counsellor	50-minute individual session provided by a counsellor	31.79	ABS [1]
	Occupational therapist	50-minute individual session provided by an occupational therapist	24.80	ABS [1]
	Nurse	50-minute individual session provided by a nurse	38.04	ABS [1]
ED presentations & hospital admissions	Inpatient psychiatric hospital admission (cost per day)	Average cost weight for DRG item codes U6** (excluding sameday admissions)	1,277.06	IHACPA [5]
	Inpatient medical hospital admission (cost per day)	Average cost weight for all DRG item codes (excluding sameday admissions)	2,064.72	IHACPA [5]
	Emergency department presentation (cost per presentation)	Average cost weight for non-admitted ED presentations	449.32	IHACPA [5]

Abbreviations: A$ - Australian dollars; ABS - Australian Bureau of Statistics; Bef - befriending; CAT - cognitive analytic therapy; DRG - Diagnosis-Related Group; ED - emergency department; IHACPA - Independent Health and Aged Care Pricing Authority; MBS - Medicare Benefits Scheme; PBS - Pharmaceutical Benefits Scheme.

Appendix Table 11 Unit cost data for psychotropic medications

Medication name	Unit cost for one gram (2015 A$)	Source
alprazolam	219.21	PBS [4]
amisulpride	5.02	PBS [4]
aripiprazole	424.40	PBS [4]
citalopram	8.44	PBS [4]
desvenlafaxine	14.14	PBS [4]
diazepam	14.02	PBS [4]
duloxetine	19.13	PBS [4]
escitalopram	18.36	PBS [4]
fluoxetine	19.41	PBS [4]
fluvoxamine	6.27	PBS [4]
mirtazapine	12.07	PBS [4]
moclobemide	0.97	PBS [4]
naproxen	0.35	PBS [4]
nitrazepam	35.56	PBS [4]
olanzapine	283.63	PBS [4]
oxazepam	5.88	PBS [4]
paliperidone	906.75	PBS [4]
paroxetine	15.77	PBS [4]
propranolol	1.99	PBS [4]
quetiapine	8.16	PBS [4]
risperidone	328.53	PBS [4]
sertraline	2.50	PBS [4]
temazepam	13.49	PBS [4]
valproic acid	0.73	PBS [4]
venlafaxine	6.18	PBS [4]
zolpidem	117.09	CW [9]
zopiclone	99.03	PBS [4]

Abbreviations: A$ - Australian dollars; CW - Chemist Warehouse; PBS - Pharmaceutical Benefits Schedule.

Appendix S6 – Summary of cost and utility weight data

Appendix Table 12 Descriptive statistics for cost data (complete cases and multiple imputation)

	HYPE+CAT			HYPE+Bef			YMHS+Bef		
**Cost parameter (2015 A$)**	**N**	**Mean**	**SE**	**N**	**Mean**	**SE**	**N**	**Mean**	**SE**
Complete cases only									
MOBY intervention	46	1,123	110	46	1,046	100	47	476	85
Other healthcare services (not related to the MOBY intervention)	20	521	166	26	544	159	24	1,146	220
ED presentations & hospital admissions	46	3,740	1,693	46	454	320	47	1,564	1,004
Medications	20	277	80	26	277	78	24	886	707
Total costs	20	5,535	2,811	26	2,275	392	24	5,619	2,177
Multiple imputation									
MOBY intervention	46	1,123	110	46	1,046	100	47	476	85
Other healthcare services (not related to the MOBY intervention)	46	497	115	46	500	111	47	1,127	187
ED presentations & hospital admissions	46	3,740	1,693	46	454	320	47	1,564	1,004
Medications	46	270	56	46	236	55	47	715	526
Total costs	46	5,629	1,765	46	2,236	383	47	3,883	1,211

Abbreviations: A$ - Australian dollars; Bef - befriending; CAT - cognitive analytic therapy; ED - emergency department; HYPE - Helping Young People Early; N - sample size; SE - standard error; YMHS - Youth Mental Health Service.

Appendix Table 13 Descriptive statistics for AQoL-8D utility weight data (complete cases and multiple imputation)

	HYPE+CAT			HYPE+Bef			YMHS+Bef		
**AQoL-8D utility weight, by follow-up**	**N**	**Mean**	**SE**	**N**	**Mean**	**SE**	**N**	**Mean**	**SE**
Complete cases only									
Baseline	46	0.341	0.023	46	0.320	0.018	47	0.298	0.016
3 months	31	0.460	0.037	40	0.415	0.031	36	0.442	0.034
6 months	32	0.521	0.043	30	0.461	0.037	34	0.490	0.040
12 months	26	0.541	0.045	32	0.590	0.043	33	0.552	0.046
18 months	22	0.636	0.053	35	0.557	0.041	29	0.590	0.051
Multiple imputation									
Baseline	46	0.341	0.023	46	0.320	0.018	47	0.298	0.016
3 months	46	0.466	0.035	46	0.413	0.030	47	0.436	0.031
6 months	46	0.513	0.041	46	0.445	0.030	47	0.474	0.035
12 months	46	0.536	0.037	46	0.570	0.038	47	0.525	0.040
18 months	46	0.623	0.043	46	0.540	0.038	47	0.562	0.044

Abbreviations: AQoL-8D - Assessment of Quality of Life-8D; Bef - befriending; CAT - cognitive analytic therapy; HYPE - Helping Young People Early; N - sample size; SE - standard error; YMHS - Youth Mental Health Service.

References

1. Australian Bureau of Statistics. 6306.0 - Employee Earnings and Hours, Australia, May 2016 [Online]. Australian Bureau of Statistics. 2017 [cited 1 June 2022]; Available from: https://www.abs.gov.au/AUSSTATS/abs@.nsf/Lookup/6306.0Main+Features1May%202016

2. Chanen AM, Betts JK, Jackson H, Cotton SM, Gleeson J, Davey CG, Thompson K, Perera S, Rayner V, Andrewes H, McCutcheon L. Effect of 3 Forms of Early Intervention for Young People With Borderline Personality Disorder: The MOBY Randomized Clinical Trial. JAMA Psychiatry 2022;79(2):109-19.

3. Services Australia. Medicare Item Reports [Online]. Australian Government. 2022 [cited 1 June 2022]; Available from: http://medicarestatistics.humanservices.gov.au/statistics/mbs_item.jsp

4. Services Australia. Pharmaceutical Benefits Schedule Item Reports [Online]. Australian Government. 2022 [cited 1 June 2022]; Available from: http://medicarestatistics.humanservices.gov.au/statistics/pbs_item.jsp

5. Independent Health and Aged Care Pricing Authority. National Hospital Cost Data Collection (NHCDC) Public Sector Report 2014–15 [Online]. IHACPA. 2017 [cited 1 June 2023]; Available from: https://www.ihacpa.gov.au/resources/national-hospital-cost-data-collection-nhcdc-public-sector-report-2014-15

6. Husereau D, Drummond M, Petrou S, Carswell C, Moher D, Greenberg D, Augustovski F, Briggs AH, Mauskopf J, Loder E, Force CT. Consolidated Health Economic Evaluation Reporting Standards (CHEERS) statement. BMJ 2013;346:f1049.

7. Sanders GD, Neumann PJ, Basu A, Brock DW, Feeny D, Krahn M, Kuntz KM, Meltzer DO, Owens DK, Prosser LA, Salomon JA, Sculpher MJ, Trikalinos TA, Russell LB, Siegel JE, Ganiats TG. Recommendations for Conduct, Methodological Practices, and Reporting of Cost-effectiveness Analyses: Second Panel on Cost-Effectiveness in Health and Medicine. JAMA 2016;316(10):1093-103.

8. Jakobsen JC, Gluud C, Wetterslev J, Winkel P. When and how should multiple imputation be used for handling missing data in randomised clinical trials - a practical guide with flowcharts. BMC Med Res Methodol 2017;17(1):162.

9. Chemist Warehouse. Chemist Warehouse: Australia's Cheapest Online Pharmacy [Online]. Chemist Warehouse. 2023 [cited 10 September 2023]; Available from: https://www.chemistwarehouse.com.au/
